# Single Channel Source Separation with ICA-Based Time-Frequency Decomposition †

**DOI:** 10.3390/s20072019

**Published:** 2020-04-03

**Authors:** Dariusz Mika, Grzegorz Budzik, Jerzy Józwik

**Affiliations:** 1 Institute of Technical Sciences and Aviation, The State School of Higher Education in Chelm, 22-100 Chelm, Poland; 2 Faculty of Mechanical Engineering, Rzeszow University of Technology, 35-959 Rzeszow, Poland; gbudzik@prz.edu.pl; 3 Faculty of Mechanical Engineering, Lublin University of Technology, 20-618 Lublin, Poland; j.jozwik@pollub.pl

**Keywords:** independent component analysis, single channel source separation, audio unmixing, clustering, sensors

## Abstract

This paper relates to the separation of single channel source signals from a single mixed signal by means of independent component analysis (ICA). The proposed idea lies in a time-frequency representation of the mixed signal and the use of ICA on spectral rows corresponding to different time intervals. In our approach, in order to reconstruct true sources, we proposed a novelty idea of grouping statistically independent time-frequency domain (*TFD*) components of the mixed signal obtained by ICA. The *TFD* components are grouped by hierarchical clustering and *k*-mean partitional clustering. The distance between *TFD* components is measured with the classical Euclidean distance and the β distance of Gaussian distribution introduced by as. In addition, the *TFD* components are grouped by minimizing the negentropy of reconstructed constituent signals. The proposed method was used to separate source signals from single audio mixes of two- and three-component signals. The separation was performed using algorithms written by the authors in Matlab. The quality of obtained separation results was evaluated by perceptual tests. The tests showed that the automated separation requires qualitative information about time-frequency characteristics of constituent signals. The best separation results were obtained with the use of the β distance of Gaussian distribution, a distance measure based on the knowledge of the statistical nature of spectra of original constituent signals of the mixed signal.

## 1. Introduction

Blind signal separation (BSS) is one of the areas of blind signal processing (BSP), a rapidly developing and very promising field of signal processing. The term “blind” refers to the fact that BPS methods make it possible to separate source signal from mixed signals without the aid of any information or training data. These methods have numerous applications in many research fields, including medical imaging and engineering [1,2,3,4], image processing and speech recognition [5,6] and communication systems [7], as well as astrophysics [8]. In audio engineering, besides speech recognition, BSS can also be used for automatic transcription or speech and musical instrument identification [9].

One of the BSS methods is independent component analysis (ICA) [10], which has gained popularity in a wide range of applications due to its conceptual simplicity and results quality. The ICA technique is a method that uses linear transformation to find statistically independent components from multidimensional mixed data (mixed multichannel signals), assuming that the source signals are statistically independent too. Examples of such multichannel data are audio or vibration signals generated by microphones or vibration sensors recording signals from different measurement points. Standard ICA consists in finding the extreme value of the cost function describing statistical independence, which means that the obtained components will be maximally statistically independent. The efficiency of ICA depends on the cost function selection and the employed optimization strategy [10].

**Figure 1:**
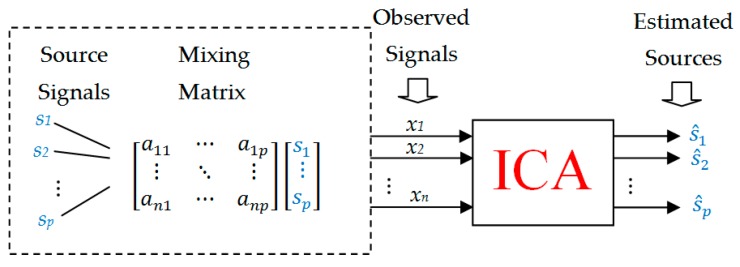
Block diagram of standard independent component analysis.

Standard ICA makes use of a multichannel signal, with the number of channels *n* (the number of microphones or sensors) not being lower than the number of source signals *p*. ICA consists in calculating statistically independent components (source signals) $s_{1},\ldots ,s_{p}$ and a p×n mixing matrix *A* for n≥p only based on *n* values of observed signals (signals generated by microphones or sensors) $x_{1},\ldots ,x_{n}$. A standard linear ICA model has the form of Equation (1):

(1) x=As

where $x={\left(x_{1},\ldots ,x_{n}\right)}^{T}$ is a vector of observed signals, $s={\left(s_{1},\ldots ,s_{p}\right)}^{T}$ is a vector of source signals, A is an n×p mixing matrix (Figure 1). The separation problem is solved by ICA as Equation (2):

(2) $$\hat{s}=Wx=WAs$$

where $\hat{s}={\left({\hat{s}}_{1},\ldots ,{\hat{s}}_{n}\right)}^{T}$ is an estimation of s and matrix W is an estimation of the inverse of A called filtration matrix. When n=p, the filtration matrix W belongs to the general linear group Gl(n) of non-singular matrices det(W)≠0.

Usually, the computational complexity of ICA is reduced at the pre-processing stage by so-called whitening the observed signal, which yields a signal z=Bx=BAs, where B is the whitening matrix characterized by unitary variance and decorrelation $C_{z}=E\left(zz^{T}\right)=I$. Assuming that for source signals $C_{s}=I$ we obtain Equation (3):

(3) $$I=C_{z}=E\left(zz^{T}\right)=BAE\left(ss^{T}\right){\left(BA\right)}^{T}=BA{\left(BA\right)}^{T}$$

This shows that ${\left(BA\right)}^{T}={\left(BA\right)}^{-1}$, or BA, is an orthogonal matrix (transformation from s to z takes place via an orthogonal matrix BA). Therefore, if $\hat{s}=Q^{T}z=Q^{T}BAs=Us$, then the matrix $U=Q^{T}BA$ is a permutation matrix, and thus a new filtering matrix Q (after whitening) must also satisfy the orthogonality condition. The solving of the ICA task (when n=p) is therefore reduced to an optimization on the orthogonal group O(n) or the special orthogonal group SO(n) when compared to the original optimization problem on the group Gl(n) (matrices W only satisfying the invertibility condition det(W)≠0). This is connected with a reduction of the degrees of freedom in the problem containing $n^{2}$ for the matrix W∈Gl(n) on $\frac{n\left(n+1\right)}{2}$ for the matrix Q∈SO(n).

Standard ICA is based on the assumption that the number of source signals $s_{i}$ is known and equal to the number of observed signals $x_{i}$, i.e., n=p. Still, the ICA estimation can also be performed for a more general case, i.e., when the number of estimated sources *p* is unknown. In this case, it is possible that n≠p. When n<p, i.e., when the number of observed signals is lower than that of source signals, we are dealing with over-complete ICA bases, but when n>p we are dealing with under-complete ICA [11,12]. From a mathematical point of view, such problem can be considered an unconstrained optimization on the Stiefel manifold [13,14,15,16,17].

Many ICA-based methods were used to separate mixed signals [18,19,20,21]. In audio engineering, observed (mixed) signals usually have the form of double channel (stereophonic) or single channel signals. In the case of a single channel signal, which is an “extremely over-complete” ICA model, Equations (1) and (2) cannot be directly employed. In the case of a stereophonic signal, which is known as the problem of under-complete ICA (n<p), differences between channels in intensity and phase of the signals are used for demixing [22,23,24,25]. Wang and Brown [26] introduced a perceptually motivated technique known as the computational auditory scene analysis (CASA) for single channel separation. Nevertheless, it must be emphasized that the effectiveness of such methods is limited and thus some additional a priori information about source signals is required. Most studies in this field are devoted to the extraction (separation) of speech signals [27,28], a commonly used approach is the so-called the W-disjoint orthogonality of signals that assumes their non-overlapping in the time-frequency plane [25,29,30]. Jang and Lee [20] proposed a single channel separation method that use the basis signals obtained by learning the probabilistic properties of sources [31]. Taghia and Doostari [32] used band-wide decomposition of mixed signal components and used ICA for signals mixed in time domain. Davies and James [33] proposed the Single Channel ICA (SCICA) method which is also based on the time domain. In [19] Casey used a single channel separation method that is based on the use of spectrograms of observed signals. In this method, the time-frequency representation of a signal (spectrogram) is treated as a multichannel observed signal and can this be separated by ICA. ICA-obtained statistically independent time-frequency components are then grouped by the Kullback–Liebler measure in order to reconstruct source signals. A similar albeit less complicated approach was adopted by Barry et al. [21]. They separate two signals by using only two spectrogram rows (spectrogram matrix) separated by 330 ms assuming additionally that spectrum of the signals was stationary over this time. A similar approach was taken by Wang and Plumbley [34]. They employed the nonnegative matrix factorisation (NMF) method on the Short Time Fourier Transform (STFT) representation of a single channel observed signal. Their algorithm, however, required the use of an additional training data. In [35], Mijovic employed both wavelet transforms and a combination of empirical mode decomposition (EMD) and ICA for ECG signals decomposition. Methods based on spectral representation of the observed signal are usually known as spectral decomposition-based methods. In [36] Litvin et al. used the bark scale aligned wavelet packet decomposition (BS-WPD) instead of the Fourier transform and at the stage of separation they use the Gaussian mixture model (GMM). In [37], Duan proposed a combination of various single channel separation methods, including some elements of the CASA, spectral decomposition based techniques and model based methods. An excellent overview of single channel source separation methods can be found in [38,39].

The paper is organized as follows. In Section 2 the proposed method of separating single-channel signals is described. There we present subsequent stages of the process and define distance measures used in the method. In addition, the use of linear ICA to solve this type of problem is also explained. In Section 3 the proposed procedure is used to signal source separation of two- and three-component mixed signals, and the quality of obtained separation is discussed in the context of the signal variance used in the analysis. Section 4 presents the results of an auditory test carried out on separated signals. Section 5 discusses the problem of computational complexity of the proposed method and offers a comparative analysis with other simple single-channel separation methods. The results of the analysis are presented in both quantitative and qualitative form. Finally, in Section 6 (Conclusions) the obtained separation results are summarized with respect to the impact of the number of source components, the spectral type of sources, as well as the impact of the signal variance used in the analysis.

## 2. Model Definition and Procedure

The proposed concept involves the use of ICA for the time-frequency t-f representation (spectrogram) of a single-channel observed signal. The representation of signal in the form of a spectrogram is actually a non-linear transformation (quadratic transformation). In this case, the use of non-linear BSS (non-linear ICA) would be appropriate. It is well known that nonlinear ICA is a difficult problem and it is generally impossible to identify unambiguously true sources [40,41]. However, under certain conditions linear ICA can be used to solve nonlinear BSS. The theoretical conditions for the use of a linear encoder, i.e., cascade PCA and linear ICA to solve a non-linear problem and reconstruct of real independent sources, are presented in [42]. Solutions are asymptotically achieved when the number of sources is high, and the numbers of inputs m (mixed signals) and non-linear bases $m_{f}$ are large relative to the number of sources $n_{s}$. In our approach, this condition is satisfied, i.e., $n_{s}=2\;\mathrm{or}\;3\ll m_{f}=m$, which means that the use of linear ICA is justified in this case.

To this end, the time signal $x_{mix}\left(t\right)$ was analysed by the Short Time Fourier Transform (STFT) in compliance with Equation (4):

(4) $$x_{mix}\left(t\right)\overset{STFT}{\to }{\mathrm{STFT}}^{mix}$$

where ${\mathrm{STFT}}^{mix}$ is the m×n complex matrix of t-f containing in m-rows instantaneous signal spectra (m is the number of STFT time frames). The input data for ICA is a spectrogram (autospectrum) of the signal $TFD^{mix}={\left|{\mathrm{STFT}}^{mix}\right|}^{2}$ [43,44]. The rows of the $TFD^{mix}$ matrix are treated as individual channels in a multichannel signal. By applying the ICA on this multichannel signal, we obtain spectral components $z_{i}$ of the t-f representation of a single channel signal which are statistically independent. The following relation holds between a $TFD^{mix}$ and matrix $Z=\left(z_{1},\ldots ,z_{m}\right)$ a matrix of statistically independent spectral components as seen in Equation (5):

(5) $$TFD_{mix}=T\cdot Z=\underset{i}{\sum }t^{i}z_{i}=\underset{i}{\sum }TFD^{i}$$

where T is a m×n mixing matrix, $t^{i}$ is an *i*-th column of T, $z_{i}$ is an *i*-th row of Z, $TFD^{i}=t^{i}z_{i}$ is an *i*-th t-f component of a mixed one-channel signal.

Throughout this paper, the components $z_{i}$ are called spectral bases whereas the columns of *T* describing time variation of $z_{i}$ are called time bases and denoted by $t^{i}$. The matrix $TFD^{i}$, which is the product of the time basis $t^{i}$ and the spectral basis $z_{i}$, is called *i*-th t-f component. By an appropriate grouping of $TFD^{i}$ bases into subgroups generating constituent components of the mixed signal, this mix can be decomposed into p components (for comparison, see Equation (1)) using Equation (6):

(6) $$TFD_{mix}=\underset{i}{\sum }TFD^{i}=\underset{j_{1}}{\sum }TFD^{j_{1}}+\underset{j_{2}}{\sum }TFD^{j_{2}}+\ldots +\underset{j_{p}}{\sum }TFD^{j_{p}}$$

where $j_{1},\ldots ,j_{p}$ are p index sets obtained by grouping $TFD^{i}$ bases.

In [45,46], the single channel signal decomposition was done by the grouping of time bases $t^{i}$ and frequency bases $z_{i}$.

For practical reason, to reduce computational complexity, it is convenient to only use the $TFD^{i}$ bases which “carry” a specified variance of the mixed signal. Assuming that in the analysis we use $\frac{\sigma \left(TFD_{\alpha mix}\right)}{\sigma \left(TFD_{mix}\right)}=\alpha \in \left(0,1\right]$ of signal variance, Equation (5) has the following form in Equation (7):

(7) $$TFD_{\alpha mix}=\underset{i_{\alpha }}{\sum }TFD^{i_{\alpha }}$$

where the index $i_{\alpha }=\left(1,\ldots ,k\right),\;k\leq n$ corresponds to the number of $TFD^{i}$ bases “carrying” α variance of the mixed signal. The selection of α determines the number $i_{\alpha }$ of $TFD^{i}$ bases that are subsequently used in ICA estimation. These bases span a subspace $TFD_{\alpha mix}$ of the primary $TFD_{mix}$ which is maximally energetic.

The grouping of bases is, in fact, a clustering process, i.e., collecting elements into clusters [47,48]. Clustering results depend on many factors, such as the employed distance measure and clustering algorithm. The distance between base components can be defined in many ways. The selection of a given distance measure type depends on many factors, including the frequency composition of signals, degree of overlapping of signals in time and frequency, the required quality of separation and frequency-related similarity of constituent signals of the mix. In the present experiment, two types of grouping were applied. The first was based on the use of clustering algorithms (hierarchical and *k*-mean clustering), while the other involved the maximization of negentropy of separated components. ICA-based single channel separation methods primarily use component grouping based on similarity in time or frequency domain. We suggest the use of a time-frequency structure to measure the similarity features in both time and spectral domain. We cluster the (TFD)^i bases using two types of distance between $TFD^{i}$ bases, i.e., the classic Euclidean distance $D_{Euk}$ and the distance $D_{\beta }$, which we call in this study as the β distance of Gaussian distribution. The Euclidean distance $D_{Euk}$ is defined as Equation (8):

(8) $$D_{Euk}\left(i,j\right)={\left||TFD^{i}-TFD^{j}|\right|}^{2}$$

where ||·|| denotes the Frobenius norm. The generalized Gaussian distribution is expressed by Equation (9) [49]:

(9) $$p\left(y|\mu ,\sigma ,\beta \right)=\frac{\omega \left(\beta \right)}{\sigma }exp\left[-c\left(\beta \right){\left|\frac{y-\mu }{\sigma }\right|}^{2/\left(1+\beta \right)}\right]$$

where μ,σ are the expected value and the standard deviation of a random variable y, respectively. The parameter β∈[−1,0] describes the type of a random variable y, i.e., its deviation from normal distribution. The parameters ω(β) and c(β) are defined by Equations (10) and (11):

(10) $$\omega \left(\beta \right)=\frac{\Gamma {\left[\frac{3}{2}\left(1+\beta \right)\right]}^{1/2}}{\left(1+\beta \right)\Gamma {\left[\frac{1}{2}\left(1+\beta \right)\right]}^{3/2}}$$

(11) $$c\left(\beta \right)={\left[\frac{\Gamma \left[\frac{3}{2}\left(1+\beta \right)\right]}{\Gamma \left[\frac{1}{2}\left(1+\beta \right)\right]}\right]}^{1/\left(1+\beta \right)}$$

where Γ is the Gamma-Euler function.

By treating a signal spectrogram as a random variable one can describe its distribution in parametric terms, i.e., it is possible to estimate the parameters μ, σ, β based on the model in Equation (9). When the source spectrograms are known, we can find the parameter $\beta_{i,org}$. The $D_{\beta }$ distance is defined as the difference between $\beta_{i,org}$ and the parameter $\beta_{i}$ characterising the spectrogram of a constituent signal reconstructed after grouping $TFD_{rec,i}=\sum_{j_{i}}TFD^{j_{i}}$ (index $j_{i}$ was defined in Equation (6)) in the following way in Equation (12):

(12) $$D_{\beta }=\left|\beta_{i,org}-\beta_{i}\left(TFD_{rec,i}\right)\right|$$

By minimizing the $D_{\beta }$ distance for individual constituent signals one can group $TFD^{i}$ bases so that the reconstructed signals are statistically as close as possible to the original signals. The $\beta_{i}$ parameter we estimated by *a posteriori* determination of the maximum of β. When observations of the random variable $y=\left\{y_{1},\ldots ,y_{N}\right\}$ are available the *a posteriori* distribution of the β parameter is given by Equation (13) [10,18]:

(13) p(β|y)∝p(y|β)p(β)

where $p\left(y|\beta \right)=\prod_{N}\frac{\omega \left(\beta \right)}{\sigma }exp\left[-c\left(\beta \right){\left|\frac{y_{N}-\mu }{\sigma }\right|}^{2/\left(1+\beta \right)}\right]$ denotes a data likelihood [18] and p(β) is an *a priori* distribution of the β parameter. The study [18] offers practical recommendations (solutions) for calculating the p(β) distribution.

The other way of grouping $TFD^{i}$ bases consists in maximizing negentropy (negative entropy) of reconstructed constituent signals $TFD_{rec,i}$. Statistically independent constituent signals have the maximum negentropy [10,50]. By finding of reconstructed constituent signals $TFD_{rec,i}=\sum_{j_{i}}TFD^{j_{i}}$ with the maximum negentropy, we group the $TFD^{i}$ bases in a correct way. The negentropy function J(y) was approximated as Equation (14) [10]:

(14) J(y)∼[E(G(y))−E(G(ν))]

where ν is the normalized Gaussian random variable (μ=0, σ=1) and G(·) is a nonlinear function of the random variable usually having the form $G\left(y\right)=\frac{1}{a}\log\cos h\left(ay\right),\;a\in \left(1,2\right)$ or $G\left(y\right)=-\exp\left(-\frac{y^{2}}{2}\right)$. This type of approximation has numerous advantages including conceptual simplicity and rapid calculation rate [10]. As a result, it is very often used as a cost function in algorithms for solving ICA problems [51]. 

## 3. Experiment

**Figure 2:**
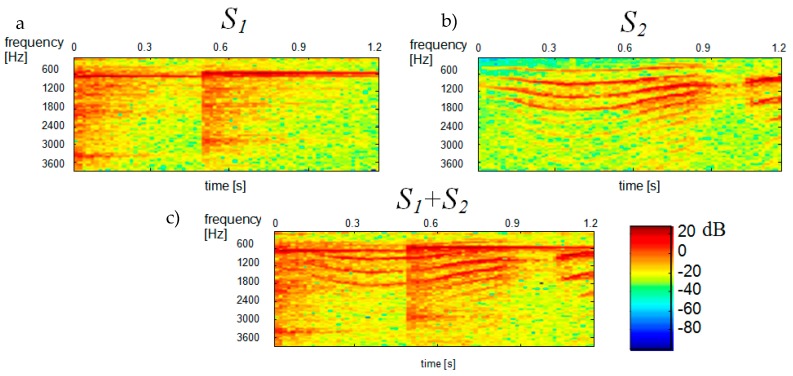
Spectrograms of constituent signals, (**a**) S1—ringer; (**b**) S2—baby and (**c**) the mixed signal S1 + S2.

The proposed idea of single channel separation was verified by experimental tests. The experiments involved demixing single-channel signal consisting of two and three constituent signals. The constituent signals $S_{1}\left(t\right)$, $S_{2}\left(t\right)$ and $S_{3}\left(t\right)$ were selected so that their spectral composition and their respective types of sources were different. The $S_{1}\left(t\right)$ signal (“ringer”) was generated by an electric device and was a recording of an electric ringer, while the $S_{2}\left(t\right)$ signal (”baby”) was a baby cry, which means that it had a specific stochastic variation of the spectre, as do all sounds generated by living beings. The $S_{3}\left(t\right)$ signal (“tom”) was a sound generated by a percussion instrument and, as such, was a typical impulsive signal. The above constituent signals were mixed in the following combinations: $S_{2mix}\left(t\right)=S_{1}\left(t\right)+S_{2}\left(t\right)$ and $S_{3mix}\left(t\right)=S_{1}\left(t\right)+S_{2}\left(t\right)+S_{3}\left(t\right)$. The signals were recorded at the sampling frequency $F_{s}=8\;\mathrm{kHz}$ and their duration was 1.2 s. Mixed single channel signal was transformed to the frequency domain using the STFT. We use blocks 256 samples long, 50% overlapped. The t-f analysis was performed in two separate blocks of 3968 and 5888 samples corresponding to the time intervals of 0–0.51 s and 0.51–1.2 s, respectively, in order to ensure higher stationarity of signal spectra in individual blocks. We used full signals of 9856 samples to determine the $D_{\beta }$ distance. Figure 2 shows the spectrograms of constituent signals $S_{1}\left(t\right)$ and $S_{2}\left(t\right)$, with the spectrogram on the left showing the $S_{1}\left(t\right)$ signal (“ringer”) and the spectrogram on the right showing the $S_{2}\left(t\right)$ signal (“baby”).

**Figure 3:**
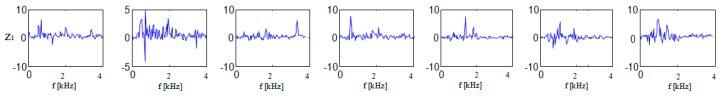
7 spectral bases $z_{i}$ obtained by ICA on the spectrogram of the mixed signal for variance α=0.85.

**Figure 4:**
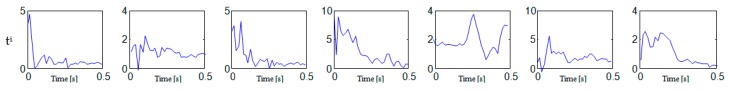
7 time bases $t^{i}$ obtained by ICA on the spectrogram of the mixed signal for variance α=0.85.

**Figure 5:**
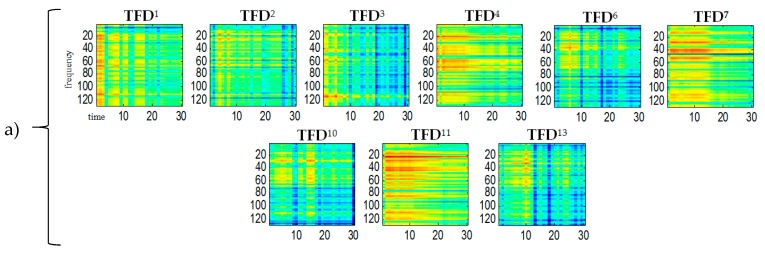

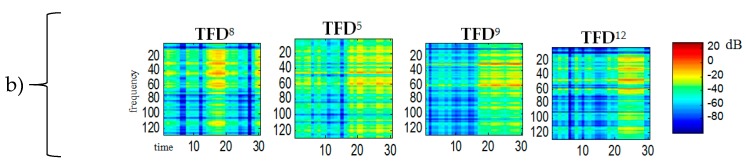
13 statistically-independent $TFD^{i}$ bases obtained by ICA on the spectrogram of the mixed signal for a signal variance α=0.9: (**a**) $TFD^{i}$ bases belonging for *S*_1_ source (**b**) $TFD^{i}$ bases belonging for *S*_2_ source.

The STFT-generated spectrogram of $TFD_{2mix}$ (bottom diagram in Figure 2) was treated as a multichannel signal and estimated by ICA. This was done using the FastICA Matlab function algorithm based on [14]. Signal whitening was performed by singular value decomposition (SVD) using the Matlab function *svd*. ICA-generated statistically independent spectral bases $z_{i}$, time bases $t^{i}$ and time-frequency bases $TFD^{i}$ for the variance α=0.85 of the input signal are shown in Figure 3, Figure 4 and Figure 5, respectively.

For all $TFD^{i}$ shown in the Figure 5 the ordinate axes scales range 0–129, which corresponds to the frequency range 0–4 kHz. The time scale range 0–30 corresponds to the range 0–0.51 s. A comparison of the obtained $TFD^{i}$ bases in Figure 2 reveals that bases 4, 7, 11 belong to the spectrogram of the *S*_1_ signal (ringer). Both this figure and some subsequent figures show the ICA results made in the first sample block (from 0 to 0.51 s).

**Figure 6:**
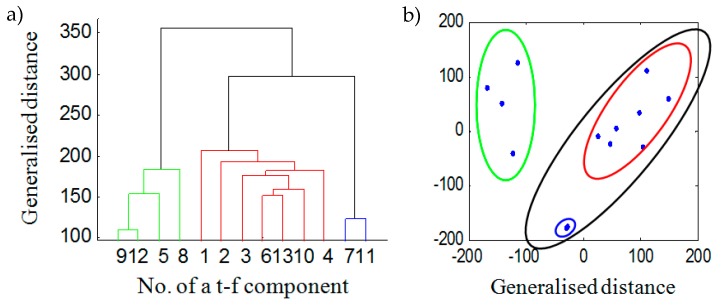
The results of hierarchical clustering for the Euclidean distance for $TFD^{i}$ components (**a**), and visualisation of groups of $TFD^{i}$ obtained by multidimensional scaling (**b**).

The clustering was performed by hierarchical [48] and *k*-mean partitional clustering [52] using two standard Matlab functions: *dendrogram* and *kmeans*. Figure 6a shows the separation results obtained with the Euclidean distance between $TFD^{i}$ components and a dendrogram obtained by hierarchical clustering. Figure 6b illustrates the “distances” between $TFD^{i}$ components obtained by multidimensional scaling [53]. Ellipses correspond to components collected in the dendrogram shown in Figure 6a. By summing the $TFD^{i}$ components grouped in Figure 6b and shown as green and black ellipses, we obtain spectrograms of two separated components seen in Equation (15):

(15) $$\begin{array}{c} TFD_{1}=\underset{j_{1}=1,2,3,4,6,7,10,11,13}{\sum }TFD^{j_{1}}\\ TFD_{2}=\underset{j_{2}=5,8,9,12}{\sum }TFD^{j_{2}} \end{array}$$

**Figure 7:**
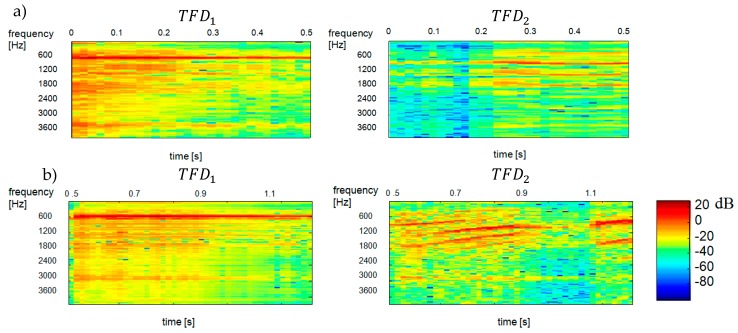
Reconstructed spectrograms (spectra) of $TFD_{1}$ and $TFD_{2}$ components as a results of hierarchical clustering with Euclidean distance for $TFD^{i}$ components. $TFD_{1}$—ringer, $TFD_{2}$—baby: (**a**) results for the time interval of 0.00–0.51 s, (**b**) results for the time interval of 0.51–1.20 s.

**Figure 8:**
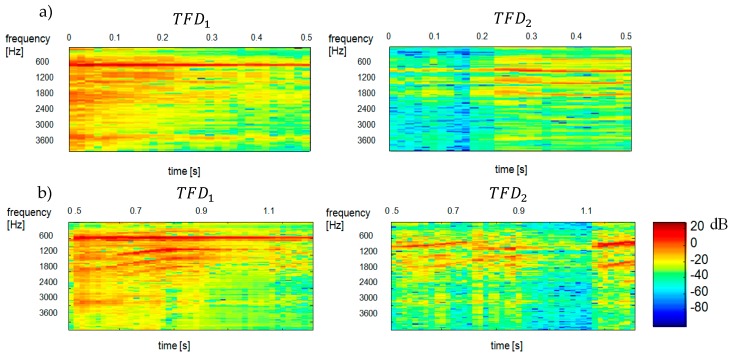
Reconstructed spectrograms (spectra) of $TFD_{1}$ and $TFD_{2}$ components obtained by minimizing the negentropy of $TFD_{1}$ and $TFD_{2}$ components. $TFD_{1}$—ringer, $TFD_{2}$—baby: (**a**) results for the time interval of 0.00–0.51 s, (**b**) results for the time interval of 0.51–1.20 s.

Figure 7 shows the reconstructed spectrograms of $TFD_{1}$ and $TFD_{2}$ components. Figure 8 shows the results of separation obtained by maximizing the negentropy of components $TFD_{1}$ and $TFD_{2}$.

**Figure 9:**
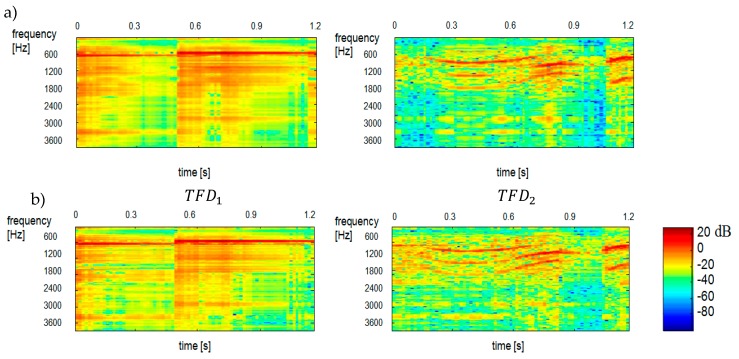
Reconstructed spectrograms (spectra) of $TFD_{1}$ and $TFD_{2}$ components obtained by k-mean partitional clustering and the β distance of Gaussian distribution. $TFD_{1}$—ringer, $TFD_{2}$—baby. The results were obtained for the variances (**a**) α=0.7 and (**b**) α=0.8, respectively, and the signal duration of 1.2 s.

An analysis of the data in Figure 9 demonstrates that the separation is effective yet it depends on the length and the variance (parameter α) of the analysed signal, and hence on the number of obtained $TFD^{i}$ bases. The lower the number of these bases is, the more effective the grouping results are obtained. Nevertheless, a decrease in the variance α results in a reduced quality of reconstruction spectrograms. The quality of separation is considerably lower for the variance α=0.7 of the mixed signal, which is manifested in the interpenetration (interference) of spectra of the constituent signals.

Figure 9 shows the results of clustering process with *β* distance of Gaussian distribution $D_{\beta }$. As it results from the presented Figure 9 results of the separation seems to be efficient. They depend however on the length of the analysed signal and the used variance value of the analysed signal (parameter α) and therefore on the number of received $TFD^{i}$ bases. The smaller the number, the better the grouping results. However, lowering the value of variance α also causes a reduction in the quality of spectrogram reconstruction. The quality of separation is significantly worse when using α=0.7 variance of the mixed signal, which is manifested by the interpenetration (interference) of spectra of the signal components.

**Figure 10:**
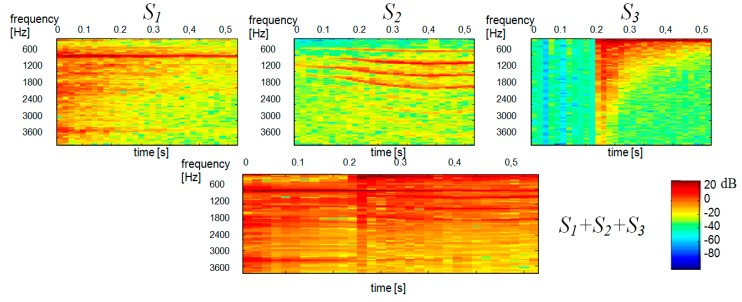
Spectrograms of constituent signals: $S_{1}$—ringer, $S_{2}$—baby, $S_{3}$—tom. The bottom spectrogram shows the mixed signal $S_{3mix}$.

**Figure 11:**
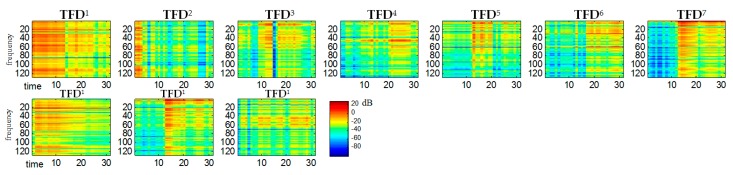
Statistically independent $TFD^{i}$ bases of a three-component signal for the variance α=0.8.

**Figure 12:**
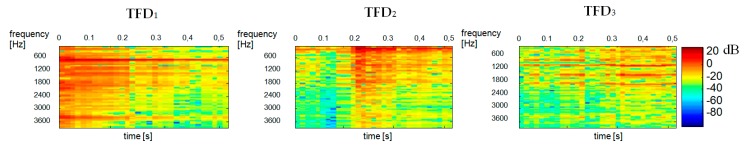
Reconstructed spectrograms of a three-component signal obtained by k-mean partitional clustering and Euclidean distance for $TFD^{i}$ bases (duration: 0.51 s): $TFD_{1}$—ringer, $TFD_{2}$—tom, $TFD_{3}$—baby.

We used our method for the demixing a single-channel signal consisting of three component signals $S_{3mix}\left(t\right)=S_{1}\left(t\right)+S_{2}\left(t\right)+S_{3}\left(t\right)$. The spectrogram of the mixed signal as well as the spectrograms of its constituent signals were shown in Figure 10. Like in Figure 5 the scales range 0–129 for all $TFD^{i}$ corresponds to the frequency range 0–4 kHz. The time scale range 0–30 corresponds to the range 0–0.51 s. Statistically independent $TFD^{i}$ bases are shown in Figure 11. One can notice a sharp similarity between $TFD^{i}$ bases and the constituent sounds of the $TFD_{i}$ mixed signal. To give an example, $TFD^{1}$, $TFD^{2}$, $TFD^{8}$ are ringer sounds, $TFD^{5}$, $TFD^{7}$ and $TFD^{9}$ are tom sounds, while other bases are baby sounds. Hence, at the clustering stage, the $TFD^{i}$ bases were grouped into 3 classes (clusters) by *k*-mean partitional clustering. Figure 12 shows the results of separation of a three-component signal. 

## 4. Perceptual Evaluation

For each of the decomposition versions presented in Section 3, the inverse STFT for every separated $TFD_{i}$ was used. The proposed separation method has been implemented in Matlab. The inverse STFT involved reconstructing time signals based on the spectrograms of separated $TFD_{i}$ bases. Given that such transformation is only based on amplitude information (spectrograms do not contain phase information), the time signals were additionally burdened with the error of “imprecise” invertibility of the STFT. In order to eliminate the effect of “imperfect” invertibility of the STFT (phase distortion), the reference signal’s sounds of the mix were also re-synthesized with zero phase. The RMS values of all separated and reference signals were normalised. All sounds were Microsoft Windows system sounds and were resampled to 8 kHz. 

For the purpose of the test, 9 pairs of reference (original) and separated sound were prepared. These pairs are called “samples”. We generated 5 sets of samples (one set per every listener), each containing 9 samples. Sequence of samples was random and different in each set. The samples were separated by 3 to 4 s of silence. Each of five participants listened to five sets of samples. The participants included one sound engineer, two instrumental musicians and two individuals not related to music. Every listener listened to samples at the same loudness (over 80 dBA) over the AKG K271 closed-back (studio) headphones in studio room. Degradation category rating scale [54] was used to rate the quality of separation by the listener. The original five-point scale was extended to six-point, as suggested by the listeners. A score of 1 means “very distorted” while a score of 6 means “inaudibly distorted”. Before the final test, each listener underwent a short training session. 

Table 1 gives the scores (mean values and standard deviations) of perceptual quality of separation with β distance of Gaussian distribution $D_{\beta }$ and the Euclidean distance for $TFD^{i}$ components. Table 2 shows the impact of the mixed signal variance used (α=0.7 or α=0.9) on the perceptual quality of separation.

The best results were obtained for the separation performed with the use of the β distance. The ringer sound was most efficiently unmixed for every mixed signal type and distance measure. The results of the baby sound are worse. The tom sound was the most difficult to separate. These results demonstrate that the proposed method is the most effective for signals (sounds) with a quasi-stationary signals with harmonic spectrum (ringer) and the least effective for non-stationary signals with a noise-like spectrum (tom). The quality of separation is higher when the variance α of the mixed signal is higher (Table 2) and, as expected, when separating from two-component mixes. In this case, specifically, the results are 0.5 points higher on the average.

## 5. Computational Complexity and Comparison Analysis

In this section, we evaluate the computational complexity of the proposed methods and compare our results with those obtained by other simple single-channel source separation methods. Our approach consists of five stages of processing: transformation of the time signal into a spectrogram, ICA stage with whitening as pre-processing, calculation of distance measure, grouping and inverse transform to the time domain. We consider the approximate number of floating point operations (flops). The code is implemented on a 2.8 GHz (CPU), 8 GHz (RAM) platform. At the transformation stage, we employ STFT with the FFT algorithm which is a very effective method because it involves overall $2n\left(log_{2}2n\right)$ (only the most significant terms are retained) flops for the time window (time segment), where *2n* is the number of samples in the time window used in STFT. Using the big O notation, the computational complexity of this stage is $O\left(n\left(log_{2}n\right)\right)$. In the ICA stage, we used the Singular Value Decomposition (SVD) as pre-processing which involves $O\left(mn^{2}\right)$ flops, where m is the number of time segments used in STFT stage. At the SVD sub-stage, we reduced the dimension of the analysis based on the desired signal variance value α. In the ICA stage, we used the FastICA algorithm which is a very effective algorithm and requires only $2\left(m_{\alpha }+1\right)n$ [55] per iteration, where $m_{\alpha }<m$ is a dimension of ICA reduced in the SVD sub-stage. This means that the approximation of complexity in the ICA stage is of order $O\left(m_{\alpha }n\right)$. In the stage of calculating the distance between the $TFD^{i}$ bases we used two types of distances: the classic Euclidean distance $D_{Euk}$ and the distance $D_{\beta }$, that require approximately $O\left(\left(\begin{array}{c} m_{\alpha }\\ 2 \end{array}\right)\cdot m_{\alpha }^{2}n^{3}\right)$ and $O\left(m_{\alpha }^{3}n^{2}\right)$ flops, respectively. In the clustering stage, we used the hierarchical clustering algorithm (single-linkage type) or the k-mean algorithm. Both algorithms have computational complexity of order $O\left({\left(mm_{\alpha }n\right)}^{2}\right)$ [48] but it includes the complexity of distances $D_{Euk}$ and $D_{\beta }$ calculating as the main stage of clustering process. At the inverse transform stage, we used IFFT algorithm which requires, similar to FFT, $O\left(n\left(log_{2}n\right)\right)$ flops.

In order to compare our method with others solutions, we additionally carry out single-channel separation using the method proposed in [19] and the method based on analysing the similarity of time bases $t^{i}$ which are called here as TFD-SCSS, KL-SCSS and T-SCSS, respectively. In the KL-SCSS method, the Kullback–Leibler distance (symmetrical Kullback–Leibler divergence) is used as a measure of distance for the spectral bases $z_{i}$. In the T-SCSS method we use the Euclidean distance for time bases $t^{i}$. Separation efficiency is measured using the root mean square error indicator (RMSE) compared to the original sources. Considering the spectrograms of the original $TFD_{org}^{i},\;i=1,2,\ldots ,n_{s}$ sources and separate $TFD^{i},\;i=1,\;2,\ldots ,n_{s}$ sources, the RMSE is calculated as:

(16) $$RMSE=\sqrt{\frac{\sum_{i}\sum_{k,l}{\left(TFD_{org}^{i}\left(k,l\right)-TFD^{i}\left(k,l\right)\right)}^{2}}{\sum_{i}\sum_{k,l}{\left(TFD_{org}^{i}\left(k,l\right)\right)}^{2}}}$$

where k,l are the row and column indices of the $TFD_{org}^{i}$ and $TFD^{i}$ indices.

The same set of source and mixed signals as in the auditory tests (Section 4) as well as the same analysis parameters are used in the comparative analysis. Table 3 presents the average results of the RMSE index for four combinations of mixed signals. It can be stated that our method based on the time and frequency domain similarity generally yields better separation results than those obtained with the methods that only use time or spectral similarity. For the mixed signal ringer + tom, better separation results are obtained using T-SCSS. This probably results from the clear differences in the time structure of the signal sources and better matching of distance in the T-SCSS method.

In addition, the time-course results are subjected to auditory testing. Table 4 gives the scores (mean values and standard deviations) of the perceptual quality of separation of our methods with the β distance of the Gaussian distribution $D_{\beta }$ and the KL-SCSS and T-SCSS methods. 

## 6. Conclusions

This study proposed a new ICA-based method for single channel separation in time-frequency domain. In terms of the grouping of $TFD^{i}$ bases and distance measure types, the methods can be divided into those which require some information about the source signals (the β distance) and those which only exploit the similarity between $TFD^{i}$ bases (Euclidean distance and negentropy minimization). The aim should be to group the bases without the use of any information about constituent signals. Nevertheless, the selection of a distance depends on the constituent signals $S_{j}\left(t\right)$, which means that some information about the mixed signal is required. If the signal amplitude varies in time to a significant extent, the Euclidean distance should be employed. This distance is by nature predisposed to group the spectral and time features of a signal. It has been shown that clustering analysis (in hierarchical and *k*-means forms) can be effectively used to group basis components of the signals. In order for the decomposition to be successful, the source components of mixed signals should have a stationary spectrum in the analysed period. Although this limitation can be overcome by shortening the analysed period, it causes in the deterioration in audible quality of reconstructed signals. The main limitation of the method is the lack of universality of the procedure. The selection of a distance measure and a clustering algorithm depends on the time-frequency structure of component signals of the mix. In addition to that, the results of separation greatly depend on the variance parameter α. If a value of α is too high and thus the number of $TFD^{i}$ bases is high too, the clustering will yield worse results. This is caused by the scattering of characteristics of the constituent signal spectra with a greater number of $TFD^{i}$ bases. On the other hand, if a value of α is too low, the quality of reconstructed signal spectra will be lower too. The quality of separation also depends on ICA limitations. As the number of mixed signals increases, the quality of separated component signals decreases, which is evidenced in the interpenetration of the component signal spectra.

## Figures and Tables

**Table 1:** Results of test in the form of mean scores and standard deviations for each sound obtained with the Euclidean distance and the β distance of Gaussian distribution $D_{\beta }$ for $TFD^{i}$ bases.

	Euclidean Distance	β Distance
baby	mean = 2.4400; σ = 0.9025	mean = 3.4160; σ = 1.1158
ringer	mean = 3.1200; σ = 1.0375	mean = 4.4480; σ = 0.9875
tom	mean = 2.5333; σ = 0.6644	mean = 3.0500; σ = 0.7961

**Table 2:** The impact of the mixed signal variance used on the perceptual quality of separation.

Measure of Distance	“Ringer”		“Baby”		“Tom”	
	α = 0.9	α = 0.7	α = 0.9	α = 0.7	α = 0.9	α = 0.7
β distance	5.00	4.36	4.32	3.04	2.75	2.14
Euclidean distance	3.44	4.00	2.44	2.64	2.42	1.63

**Table 3:** RMSE index mean and standard deviation for separation algorithms used in comparative analysis.

Separation Algorithm	RMSE (Mean and Std. dev.)			
	Baby + Ringer	Ringer + Tom	Baby + Tom	Baby + Ringer + Tom
TFD-SCSS	0.2120 ± 0.0235	0.1138 ± 0.0134	0.1821 ± 0.0148	0.3125 ± 0.0725
KL-SCSS	0.2935 ± 0.0455	0.3120 ± 0.0436	0.3120 ± 0.0445	0.5120 ± 0.1215
T-SCSS	0.2330 ± 0.0145	0.0820 ± 0.0212	0.2020 ± 0.0135	0.3520 ± 0.0935

**Table 4:** Results of test in the form of mean scores and standard deviations for analysed methods.

Source Signals	TFD-SCSS	KL-SCSS	T-SCSS
baby	mean = 3.4160; σ = 1.1158	mean = 2.8420; σ = 1.3457	mean = 3.2180; σ = 1.3651
ringer	mean = 4.4480; σ = 0.9875	mean = 3.8490; σ = 0.9961	mean = 4.5460; σ = 0.9354
tom	mean = 3.0500; σ = 0.7961	mean = 2.7533; σ = 0.9832	mean = 2.9544; σ = 0.8794
